# 221. Assessment and Proposed Revision of Clinical Trial *Clostridioides difficile* Infection Clinical Response and Outcomes Definitions

**DOI:** 10.1093/ofid/ofac492.299

**Published:** 2022-12-15

**Authors:** Anne J Gonzales-Luna, Andrew M Skinner, Carolyn D Alonso, Oliver A Cornely, Kevin W Garey, Dale N Gerding, Stuart Johnson, Stacy A Kahn, Ciaran P Kelly, Colleen R Kelly, Larry K Kociolek, Ed J Kuijper, Ed J Kuijper, Thomas J Louie, Thomas V Riley, Thomas J Sandora, Maria Vehreschild, Mark H Wilcox, Erik R Dubberke

**Affiliations:** University of Houston, Houston, Texas; Loyola University Chicago Stritch School of Medicine, Maywood, Illinois; Beth Israel Deaconess Medical Center, Boston, Massachusetts; University of Cologne, Faculty of Medicine and University Hospital Cologne, Cologne, Nordrhein-Westfalen, Germany; University of Houston College of Pharmacy, Houston, Texas; Edward Hines, Jr. Veterans Affairs Hospital, Hines, Illinois; Hines VA Hospital and Loyola University Medical Center, Hines, Illinois; Boston Children’s Hospital and Harvard Medical School, Boston, Massachusetts; Beth Israel Deaconess Medical Center, Boston, Massachusetts; Warren Alpert Medical School of Brown University, Providence, Rhode Island; Ann & Robert H. Lurie Children’s Hospital of Chicago, Chicago, Illinois; Leiden University Medical Center and RIVM, Leiden, Zuid-Holland, Netherlands; Leiden University Medical Center and RIVM, Leiden, Zuid-Holland, Netherlands; University of Calgary, Calgary, Alberta, Canada; The University of Western Australia, Nedlands, Western Australia, Australia; Boston Children's Hospital, Boston, Massachusetts; Department of Internal Medicine, Infectious Diseases, University Hospital Frankfurt, Goethe University Frankfurt, Frankfurt am Main, Germany, Frankfurt, Hessen, Germany; University of Leeds; Leeds Teaching Hospitals NHS Trust, Leeds, England, United Kingdom; Washington University, Saint Louis, Missouri

## Abstract

**Background:**

*Clostridioides difficile* infection (CDI) research is limited by a lack of standardized definitions for clinical response and disease outcomes, which impacts clinical drug development and results comparison between studies. We aimed to assess outcome definitions in CDI therapeutic trials to propose new versions that are clinically relevant, discrete and objective.

**Methods:**

A multidisciplinary group of CDI experts met monthly to review response endpoints from published clinical trials of antibiotic therapy for CDI. Previously published phase III or IV trials were assessed for outcome definitions. Discussions were held to reach a consensus on new clinical trial endpoints for adults and children to improve the accuracy and clinical relevance of measures of treatment success.

**Results:**

Significant heterogeneity was noted amongst the primary endpoints in phase III and IV CDI antibiotic treatment trials. Initial clinical cure (ICC), strictly defined as < 3 unformed bowel movements/24 hour, and sustained clinical cure (SCC) were primary outcome measures for recent clinical trials. The strict ICC definition incompletely measures treatment success as assessed in clinical practice and, since ICC is necessary to achieve SCC, may lead to type II error for SCC. A set of proposed alternative outcome definitions was developed using the terms initial response (IR) and sustained response (SR) (Figure 1). IR allows for investigator assessment of overall improvement in CDI response more analogous to clinical practice and will lead to more patients eligible to meet SR. Achievement of SR requires both IR and no need for retreatment of CDI by day 30 after antibiotic completion and is the more relevant endpoint for CDI therapeutic development. The use of a less restrictive IR definition will more accurately capture early responses to treatment and importantly increase the validity of SR. The shortening of follow-up period by 30 days is also anticipated to reduce costs and efforts associated with conducting trials.

Timeline of CDI outcome assessments for clinical trials

**Figure:**
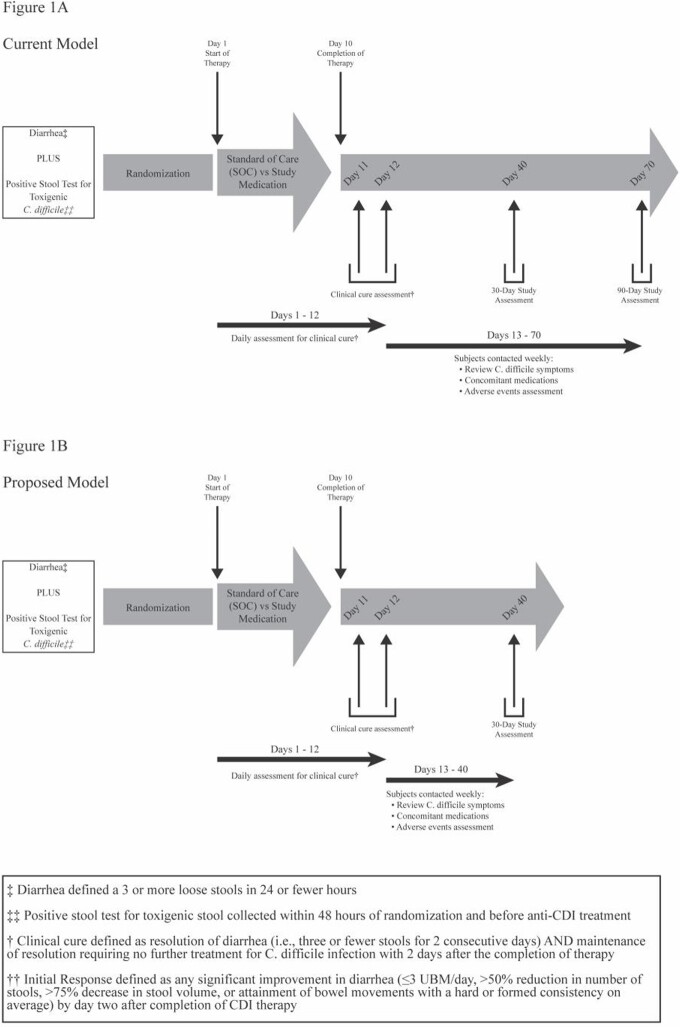

**Conclusion:**

The set of definitions proposed here will more accurately capture clinical success and standardize the approach to outcome assessment in trials of CDI therapeutics.

**Disclosures:**

**Carolyn D. Alonso, MD**, Cidara Therapeutics: Advisor/Consultant|Merck: Advisor/Consultant **Oliver A. Cornely, Prof. Dr.**, Abbott: Honoraria|Abbvie: Advisor/Consultant|Actelion: Board Member|Al-Jazeera Pharmaceuticals: Honoraria|Allecra Therapeutics: Board Member|Amplyx: Advisor/Consultant|Amplyx: Grant/Research Support|Astellas: Honoraria|Basilea: Advisor/Consultant|Basilea: Grant/Research Support|Biocon: Advisor/Consultant|Biosys: Advisor/Consultant|BMBF: Grant/Research Support|Cidara: Advisor/Consultant|Cidara: Board Member|Cidara: Expert Testimony|Cidara: Grant/Research Support|CoRe Consulting: Stocks/Bonds|Da Volterra: Advisor/Consultant|DLR: Grant/Research Support|DZIF: Grant/Research Support|Entasis: Board Member|EU Directorate-General for Resarch and Innovation: Grant/Research Support|F2G: Grant/Research Support|German Patent and Trade Mark Office: German patent (DE 10 2021 113 007.7)|Gilead: Advisor/Consultant|Gilead: Grant/Research Support|Grupo Biotoscana/United Medical/Knight: Honoraria|Hikma: Honoraria|IQVIA: Board Member|Janssen: Board Member|Matinas: Advisor/Consultant|Matinas: Grant/Research Support|MedPace: Advisor/Consultant|MedPace: Grant/Research Support|MedScape: Honoraria|MedUpdate: Honoraria|Menarini: Advisor/Consultant|Merck/MSD: Grant/Research Support|Merck/MSD: Honoraria|Molecular Partners: Advisor/Consultant|MSG-ERC: Advisor/Consultant|Mundipharma: Grant/Research Support|Mylan: Honoraria|Noxxon: Advisor/Consultant|Octapharma: Advisor/Consultant|Octapharma: Grant/Research Support|Paratek: Board Member|Pardes: Advisor/Consultant|Pfizer: Grant/Research Support|Pfizer: Honoraria|Projektträger Jülich: Grant/Research Support|PSI: Advisor/Consultant|PSI: Board Member|Pulmocide: Board Member|Scynexis: Advisor/Consultant|Scynexis: Grant/Research Support|Seres: Advisor/Consultant|Shionogi: Board Member|Wiley (Blackwell): Editor-in-Chief, Mycoses **Kevin W. Garey, PharmD, MS**, Acurx Pharmaceuticals: Grant/Research Support|Paratek Pharmaceuticals: Grant/Research Support|Seres Therapeutics: Grant/Research Support|Summit Pharmaceuticals: Grant/Research Support **Dale N. Gerding, MD**, Destiny Pharma plc.: Advisor/Consultant **Stuart Johnson, M.D.**, Ferring Pharmaceuticals: Membership on Ferring Publication Steering Committee|Ferring Pharmaceuticals: Employee|Summit Plc: Advisor/Consultant **Stacy A. Kahn, MD**, Lilly: Stocks/Bonds **Ciaran P. Kelly, n/a**, Artugen: Advisor/Consultant|Facile Therapeutics: Advisor/Consultant|Ferring Pharma: Advisor/Consultant|Finch: Advisor/Consultant|Finch: Advisor/Consultant|First Light Biosciences: Advisor/Consultant|First Light Biosciences: Ownership Interest|Milky Way Biosciences: Advisor/Consultant|Milky Way Biosciences: Grant/Research Support|Pfizer: Advisor/Consultant|Seres Therapeutics: Advisor/Consultant|Summit Therapeutics: Advisor/Consultant **Larry K. Kociolek, MD, MSCI**, Merck: Grant/Research Support **Thomas J. Louie, MD**, Artugen: Advisor/Consultant|Artugen: Grant/Research Support|Crestone: Advisor/Consultant|Crestone: Grant/Research Support|Finch Therapeutics: Advisor/Consultant|Finch Therapeutics: Grant/Research Support|Rebiotix: Advisor/Consultant|Rebiotix: Grant/Research Support|Seres Therapeutics: Advisor/Consultant|Seres Therapeutics: Grant/Research Support|summit plc: Advisor/Consultant|summit plc: Grant/Research Support|Vedanta Biosciences: Advisor/Consultant|Vedanta Biosciences: Grant/Research Support **Maria Vehreschild, Prof. Dr.**, 3M: speaker fee|Astellas: Advisor/Consultant|Astellas: speaker fee|biologische heilmittel heel gmbh: Grant/Research Support|BioNtech: Grant/Research Support|EUMEDICA: Advisor/Consultant|Farmak International Holding: Advisor/Consultant|Ferring: Advisor/Consultant|Ferring: Speaker fee|Gilead Sciences: Advisor/Consultant|Immunic AG: Advisor/Consultant|MaaT: Advisor/Consultant|Merck: Advisor/Consultant|Merck: speaker fee|MSD: Advisor/Consultant|MSD: Grant/Research Support|MSD: speaker fees|Pfizer: speaker fee|Roche Molecular Systems: Grant/Research Support|Roche Molecular Systems: speaker fees|SocraRTec R&D GmbH: Advisor/Consultant|Takeda California: Grant/Research Support **Professor Mark H. Wilcox, MD, FRCPath**, GSK: Advisor/Consultant|GSK: Board Member|GSK: Grant/Research Support|Pfizer: Advisor/Consultant|Phico Therapeutics: Board Member|Seres: Advisor/Consultant|Seres: Board Member|Seres: Grant/Research Support|Summit: Advisor/Consultant|Summit: Grant/Research Support **Erik R. Dubberke, MD, MSPH**, Abbott: Advisor/Consultant|Ferring: Advisor/Consultant|Ferring: Grant/Research Support|Merck: Advisor/Consultant|Pfizer: Advisor/Consultant|Pfizer: Grant/Research Support|Seres: Advisor/Consultant|Summit: Advisor/Consultant|Synthetic Biologics: Grant/Research Support.

